# Oral manifestations in young adults infected with COVID-19 and impact of smoking: a multi-country cross-sectional study

**DOI:** 10.7717/peerj.13555

**Published:** 2022-07-15

**Authors:** Maha El Tantawi, Heba Jafar Sabbagh, Nada Abubakor Alkhateeb, Maryam Quritum, Joud Abourdan, Nafeesa Qureshi, Shabnum Qureshi, Ahmed Hamoud, Nada Mahmoud, Ruba Odeh, Nuraldeen Maher Al-Khanati, Rawiah Jaber, Abdulrahman Loaie Balkhoyor, Mohammed Shabi, Morenike O. Folayan, Noha Gomaa, Raqiya Al_Nahdi, Nawal Mahmoud, Hanane El Wazziki, Manal Alnaas, Bahia Samodien, Rawa Mahmoud, Nour Abu Assab, Sherin Saad, Sondos Al-Hachim, Ali Alshaikh, Wafaa Abdelaziz

**Affiliations:** 1 Department of Pediatric Dentistry and Dental Public Health, Faculty of Dentistry, Alexandria University, Alexandria, Egypt; 2 Department of Pediatric Dentistry, Faculty of Dentistry, King Abdulaziz University, Jeddah, Saudi Arabia; 3 Faculty of Medicine, King Abdulaziz University, Jeddah, Saudi Arabia; 4 Medical Faculty, Istanbul Medipol University, Istanbul, Turkey; 5 City Quay Dental Practice and Implant Centre, Dundee, Scotland, United Kingdom; 6 Department of Education, University of Kashmir, Srinagar, Kashmir, India; 7 Faculty of Dentistry, Alexandria University, Alexandria, Egypt; 8 Faculty of Dentistry, National Ribat University, Khartoum, Sudan; 9 College of Dentistry, Ajman University, Ajman, United Arab Emirates; 10 Department of Oral and Maxillofacial Surgery/Faculty of Dentistry, Syrian Private University, Damascus, Syria; 11 General Courses, King Abdulaziz University, Jeddah, Saudi Arabia; 12 Faculty of Engineering, King Abdulaziz University, Jeddah, Saudi Arabia; 13 King Saud bin Abdulaziz University for Health Sciences, Riyadh, Saudi Arabia; 14 Obafemi Awolowo University, Ile-ife, Osun State, Nigeria; 15 Schulich School of Medicine and Dentistry, University of Western Ontario, Ontario, Canada; 16 Department of Dental Surgery, Oman Dental College, Muscat, Oman; 17 UCSI University, Kuala Lumpur, Malaysia; 18 Department of Cereal Plant Pathology, National Institute of Aricultural Research, Settat, Morocco; 19 Division of Imaging Science and Technology, School of Medicine, University of Dundee, Dundee, Scotland, United Kingdom; 20 Western Cape Education Department, Western Cape, South Africa; 21 International Medical Center, Jeddah, Saudi Arabia; 22 Schools of Awqaf, Directorate of Education, Jerusalem, Palestine; 23 Gothenburg University, Gothenburg, Sweden; 24 Health Education Services, Ingham County, Lansing, Michigan, United States of America; 25 Faculty of Dentistry, King Abdulaziz University, Jeddah, Saudi Arabia

**Keywords:** COVID-19, Oral lesions, Smoking, Dry mouth

## Abstract

**Background:**

Oral manifestations and lesions could adversely impact the quality of people’s lives. COVID-19 infection may interact with smoking and the impact on oral manifestations is yet to be discovered.

**Objectives:**

The aim of this study was to assess the self-reported presence of oral lesions by COVID-19-infected young adults and the differences in the association between oral lesions and COVID-19 infection in smokers and non-smokers.

**Methods:**

This cross-sectional multi-country study recruited 18-to-23-year-old adults. A validated questionnaire was used to collect data on COVID-19-infection status, smoking and the presence of oral lesions (dry mouth, change in taste, and others) using an online platform. Multi-level logistic regression was used to assess the associations between the oral lesions and COVID-19 infection; the modifying effect of smoking on the associations.

**Results:**

Data was available from 5,342 respondents from 43 countries. Of these, 8.1% reported COVID-19-infection, 42.7% had oral manifestations and 12.3% were smokers. A significantly greater percentage of participants with COVID-19-infection reported dry mouth and change in taste than non-infected participants. Dry mouth (11.1% *vs* 7.5%, *p* = 0.009) and change in taste (11.5% *vs* 2.7%, *p* < 0.001) were greater in COVID-19 infected than non-infected persons. The association between COVID-19-infection and dry mouth was stronger among smokers than non-smokers (AOR = 1.26 and 1.03, *p* = 0.09) while the association with change in taste was stronger among non-smokers (AOR = 1.22 and 1.13, *p* = 0.86).

**Conclusion:**

Dry mouth and changed taste may be used as an indicator for COVID-19 infection in low COVID-19-testing environments. Smoking may modify the association between some oral lesions and COVID-19-infection.

## Introduction

The COVID-19 pandemic has adversely affected people’s lives and caused multiple clinical manifestations (Chen et al., 2020; Zhu et al., 2020; Vinayachandran & Balasubramanian, 2021) including oral manifestations and lesions (Huang et al., 2021). Oral lesions associated with COVID-19 infection include blisters, ulcers, plaques, macule and erosion (Al-Khanati et al., 2020; Amorim dos Santos et al., 2021). A cross sectional study of COVID-19 infected patients indicated that they had aphthous-like ulcers, erythema and lichen planus (Fidan, Koyuncu & Akin, 2021). In addition, Katz & Yue (2021) reported an increased tendency for COVID-19 infection among patients with recurrent aphthous stomatitis. These manifestations could affect the quality of life of people and cause physical disability, functional limitation, and psychological disabilities (Villanueva-Vilchis et al., 2016).

Furthermore, the public health response to COVID-19 pandemic affected the mental health of many individuals because of the induced psychological distress resulting from the loss of job, uncertainties about income, prolonged isolation and poor access to social support (Kumar Kalidoss & Singh Bakshi, 2020). Shutdown of schools and poor access to support systems and movement restrictions created further stresses for many young adults. Stress is a risk factor for tobacco use (Ansell et al., 2012).

Tobacco use causes oral manifestations due to the harmful chemicals and nicotine released when tobacco is burned in the oral cavity (Ozturk, Fidanci & Unal, 2017). Tobacco smokers have oral manifestations like change in taste, hairy tongue, sore and dry throat and leukoplakia. In addition, gingival inflammation and ulcers or burns were more frequently reported in smokers than non-smokers (Patil, Bathi & Chaudhari, 2013; Amadori et al., 2017; Kumar Kalidoss & Singh Bakshi, 2020). Also, the severity of COVID-19 was reported to more severe in smokers (Gülsen et al., 2020) indicating a possible a relationship between smoking, COVID-19 infection and oral manifestations.

There is growing evidence about the association between COVID-19 and the occurrence of oral manifestations (Soares et al., 2020; Abubakr, Salem & Kamel, 2021; Fidan, Koyuncu & Akin, 2021). There are also indications that there is an increase in the proportion of people who started to smoke or increased the frequency of smoking during the pandemic (Gaiha, Cheng & Halpern-Felsher, 2020; García-Álvarez et al., 2020). There is, however, limited information on the relationship between COVID-19, smoking and oral manifestations, especially among young patients. The compromised immune system resulting from COVID-19 and the side effect of its treatment may be associated with oral lesions such as opportunistic infections of the salivary glands, dry mouth, oral ulcerations and gingivitis (Dziedzic & Wojtyczka, 2021).

Therefore, the aim of this study was to determine the association between the self-reported presence of oral manifestations in young adults from several countries and COVID-19 infection, and how this association may be modified by smoking. The null hypothesis of the study is that there is no significant difference in the self-reported presence of oral manifestations between participants infected and not infected with COVID-19, and that this difference is not modified by smoking status.

## Materials and Methods

This was a cross-sectional study that collected data from 43 countries between August 2020 to January 2021 (Table S1). Ethical approval was obtained from the Research Ethics Committee of the Faculty of Dentistry, King Abdulaziz University in Saudi Arabia (#13-08-20). The study was carried out according to the Helsinki Declaration (WMA, 2001). Participants indicated consent by returning a filled questionnaire on the electronic link. Once they accessed the link, they could agree to participate, where the link takes them to the questionnaire, or not agree, where the link will exit them from the survey. Study participants were informed about the purpose of the study and the freedom to withdraw at any time and were assured of confidentiality. There was no reimbursement for study participation.

The sample size for the study was estimated to address the study aims. For the first aim, we assumed a prevalence of oral manifestations ranging from 5% to 90% in different countries, with 5% margin of error and 95% confidence level. The required sample size ranged from 73 to 383. This was increased to 167 and 460 to accommodate a non-response rate of 20% (Select Statistical Services, 2020). For the second aim where a regression analysis was to be conducted, 500 participants were needed (Nemes et al., 2009). Thus, at least 500 participants were targeted. Recruitment continued after reaching this number to ensure maximum geographic representation.

Participants were included in the study if they were young adults aged 18–23-year-old and consented to participate. Participants had to be able to read the language of the survey and have access to an electronic device connected to the internet to be able to respond to the survey.

The questionnaire was constructed in Arabic and translated by native dentists into French, Malay, Turkish and English. The content validity index (CVI) was calculated (Yusoff, 2019) for each version of the questionnaire. In addition, each version was pilot-tested by 10 participants to ensure clarity and suitability of terms. The questionnaire was uploaded to SurveyMonkey® and a tailored link was made for each data collector. All questions were mandatory; the questionnaire did not allow to leave any blank responses.

The core team (King Abdulaziz University, KAU, Saudi Arabia, and Alexandria University, Egypt) invited collaborators to recruit data from their countries/regions. Each collaborator received the study protocol, the ethical approval in addition to the customized link and details about required number of participants. We used a snowball sampling technique where country collaborators asked individuals in their network to further circulate the questionnaire link among those they knew.

Data were collected using an electronic questionnaire that comprised two sections: the first section asked about the participants’ gender, medical problems the participant had, their COVID-19 status (confirmed COVID-19 infection based on antigen or PCR test), and if they smoked. The second section asked if participants had any of the eight listed oral lesion or conditions including dental caries, stained teeth, gingival inflammation, dry mouth, change in taste, leukoplakia, burns or ulcers, or hairy tongue.

### Statistical analysis

The associations between oral manifestations and COVID-19 and smoking status were assessed using the chi square test. Multilevel binary logistic regression was used to assess the relationship between each oral lesion and COVID-19 infection status, adjusted for gender, history of medical problems and smoking. Country of residence was entered into the model as random effect variable. Multilevel binary logistic regression was also used to assess whether smoking modified the association between oral manifestations and COVID-19 infection status after adjusting for gender and a history of medical problems with country entered as random effect variable. IBM SPSS for Windows version 22.0 (IBM Corp., Armonk, NY, USA) was used for statistical analysis. Adjusted odds ratios (AOR), confidence intervals (CI) and *p* values were calculated. Significance was set at the 5% level.

## Results

The CVI for the English and Arabic versions of the questionnaire was 0.87 based on feedback from nine dentists, 0.97 for the Turkish version (seven dentists), 0.80 for the Malay version (five dentists) and 0.88 for the French version (five dentists).

Complete responses were available from 5,342 young adults from 43 countries (Table S1). Table 1 shows that 58.5% of participants were females, 13.4% had medical problems, 8.1% reported COVID-19 infection and 12.3% were smokers.

**Table 1:** Gender, medical problems, COVID-19 infection and smoking status of 18–23 year old persons in 43 countries (*n* = 5,342).

Factors		N (%)
Gender	Male	2,219 (41.5)
	Female	3,123 (58.5)
Has medical problems	Yes	716 (13.4)
	No	4,626 (86.6)
Infected with COVID-19	Yes	434 (8.1)
	No	4,908 (91.9)
Current smokers	Yes	657 (12.3)
	No	4,685 (87.7)

Table 2 shows that 42.7% of participants reported oral manifestations. A significantly higher proportion of participants with COVID-19 infection than those without COVID-19 infection had of dry mouth, change in taste, leukoplakia, and hairy tongue (*p* < 0.05). A significantly higher proportion of participants who were smokers than those who are not smokers had stained teeth, gingival inflammation, dry mouth, change in taste, leukoplakia, burns or ulcers and hairy tongue (*p* < 0.05).

**Table 2:** Association between the presence of oral lesions and COVID-19 infection and smoking status (*n* = 5,342).

Reported oral lesions	COVID-19 infection			Smoking			Total *n* (%)
	Yes *n* (%)	No *n* (%)	*p* value	Yes *n* (%)	No *n* (%)	*p* value	
Nothing	209 (48.2)	2,854 (58.1)	<0.001	232 (35.3)	2,831 (60.4)	<0.001	3,063 (57.3)
Stained teeth	77 (17.7)	750 (15.3)	0.17	243 (37.0)	584 (12.5)	<0.001	827 (15.5)
Dental caries	103 (23.7)	1,046 (21.3)	0.24	156 (23.7)	993 (21.2)	0.14	1,149 (21.5)
Gingival inflammation	57 (13.1)	525 (10.7)	0.12	107 (16.3)	475 (10.1)	<0.001	582 (10.9)
Dry mouth	48 (11.1)	370 (7.5)	0.009	122 (18.6)	296 (6.3)	<0.001	418 (7.8)
Change in taste	50 (11.5)	131 (2.7)	<0.001	55 (8.4)	126 (2.7)	<0.001	181 (3.4)
Leukoplakia	20 (4.6)	93 (1.9)	<0.001	25 (3.8)	88 (1.9)	0.001	113 (2.1)
Burns or ulcers	10 (2.3)	59 (1.2)	0.07	21 (3.2)	48 (1.0)	<0.001	69 (1.3)
Hairy tongue	10 (2.3)	35 (0.7)	0.001	13 (2.0)	32 (0.7)	0.001	45 (0.8)

Table 3 shows the association between oral manifestations and COVID-19 infection. There were higher odds of reporting the presence of dry mouth (AOR = 1.03, 95% CI [0.82–1.29]), change in taste (AOR = 1.16, 95% CI [0.93–1.46]), leukoplakia (AOR = 1.05, 95% CI [0.83–1.33]), and hairy tongue (AOR = 1.03, 95% CI [0.81–1.30]) among those with COVID-19 infection, although none of these associations was statistically significant.

**Table 3:** Association between oral lesions and COVID-19 infection and differences due to effect modification by smoking status using multi-level binary logistic regression.

Reported presence of oral lesions	All AOR (95% CI)	Smokers AOR (95% CI)	Non-smokers AOR (95% CI)	*p* of difference due to effect modification
Stained teeth	0.95 [0.71–1.26]	1.24 [0.74–2.07]	1.00 [0.79–1.26]	0.02*
Dental caries	0.99 [0.79–1.23]	1.13 [0.66–1.92]	1.03 [0.82–1.29]	0.59
Gingival inflammation	1.00 [0.79–1.25]	1.21 [0.71–2.07]	1.02 [0.81–1.28]	0.29
Dry mouth	1.03 [0.82–1.29]	1.26 [0.74–2.15]	1.03 [0.82–1.31]	0.09
Change in taste	1.16 [0.93–1.46]	1.13 [0.65–1.98]	1.22 [0.96–1.54]	0.86
Leukoplakia	1.05 [0.83–1.33]	1.06 [0.60–1.89]	1.06 [0.83–1.35]	0.90
Burns and ulcers	1.01 [0.80–1.28]	1.00 [0.56–1.79]	1.03 [0.81–1.31]	0.95
Hairy tongue	1.03 [0.81–1.30]	1.06 [0.60–1.90]	1.03 [0.81–1.31]	0.85

**Notes:**

An asterisk (*) indicates statistically significant at *p* < 0.05. Overall model was adjusted for gender, having medical problems and smoking status with country entered as random effect variable.

AOR, adjusted odds ratio; CI, confidence interval.

Table 3 also shows the effect modification in the association between oral manifestations and COVID-19 infection by smoking status presented by differences in the adjusted odds ratios between smokers and non-smokers. Smoking significantly modified the association between self-reported presence of stained teeth and COVID-19 infection with statistically significant difference between smokers and non-smokers in the strength of the association (*p* = 0.02).

For the other oral manifestations, the odds for the association between the presence of manifestations and COVID-19 infection were higher among smokers than non-smokers although the effect modification was not significant (*p* > 0.05) except for the association between COVID-19 infection and change in taste where the association was non-significantly (*p* = 0.86) stronger among non-smokers than smokers.

## Discussion

The study showed that persons with COVID-19 infection had higher frequency of reporting dry mouth, change in taste, leukoplakia, and hairy tongue. However, after controlling for gender, having medical problems and smoking status, none of these associations was statistically significant, and the strongest association among them was for the change in taste. This suggests that underlying medical conditions and smoking may predispose persons infected with COVID-19 to these oral conditions accounting at least partly for the strength of the association between COVID-19 infection and the presence of oral manifestations or condition. Smoking significantly modified the association between COVID-19 infection and stained teeth where a directly proportional association was observed only among smokers and there was no association among non-smokers. The association between COVID-19 infection and dry mouth was stronger in smokers than in non-smokers whereas the association with change in taste was stronger among non-smokers than smokers. The null hypothesis can thus be rejected.

Our findings have implications for identifying oral manifestations associated with COVID-19 and the detection of COVID-19 infection among young adults especially in settings where the uptake of COVID-19 testing is low. In these settings, dentist’s screening for oral manifestations should have a high index of suspicion for COVID-19 when adolescents and young persons who smoke or have health problems, have oral the reported manifestations.

Similar to prior studies, we observed salivary gland-related oral manifestations such as dry mouth, and taste alteration in COVID-19 infected patients (Parma et al., 2020; Passarelli et al., 2020; Sakalli et al., 2020). The salivary gland-related oral manifestations may have resulted from the invasion of human cells by SAR-CoV-2 by binding to the angiotensin-converting enzyme two receptors in the salivary glands and on the tongue surface thereby causing inflammatory response (Xu et al., 2020).

The higher odds for a change in taste in association with COVID-19 infection than the other oral manifestations corroborate the results of a systematic review that showed a significant association between taste disorders and COVID-19 infection (Amorim dos Santos et al., 2021). This taste alteration was attributed to local inflammation of the taste buds, peripheral neurotropism and direct toxicity to taste buds, and to side effects of COVID treatment (Finsterer & Stollberger, 2020; Mahmoud et al., 2021).

The study showed that smoking modified the association between COVID-19 infection and teeth staining where the odds ratios were significantly higher in smokers than in non-smokers although these associations were not statistically significant. Nicotine significantly increases the number of *Streptococcus mutans* in the mouth of smokers hence promoting the formation of dental plaque that gets stained by cigarette contents (Liu et al., 2018). Smokers also had higher odds of having dry mouth than non-smokers though this finding was not significant as reported in prior studies (Villa et al., 2011; Andersson & Johannsen, 2016). Unlike prior studies, we provide evidence about this association in young people and not just adults. The results of the regression analysis, however, indicate that dry mouth association with COVID-19 may be modified by smoking.

We also observed that the association between alteration in taste and COVID-19 infection was greater in non-smokers than smokers. COVID-19 infection may cause chemosensory impairment that affects taste sensation (Parma et al., 2020), and smoking may have been associated with a lower likelihood of an alteration in taste happening for smokers as smoking already blunts the perception of flavors and elevates the taste threshold (Da Ré et al., 2018) especially the taste of salt and sugar (Da Ré et al., 2018; Duffy et al., 2019). Change in taste is becoming recognized as a COVID-19 associated manifestation. Smokers need to be warned that they may not experience this manifestation as non-smokers and should avoid a possible false sense of security against catching COVID-19 infection because of this.

The present study has some limitations. First, its cross-sectional design cannot prove causation but only suggest association. Second, self-reporting may under or overestimate the presence of some oral manifestations, although this may be the only method to assess the presence of some conditions such as change in taste. Also, a recent study compared clinical evaluation with self-reported oral conditions and showed a positive association between both reports (Nascimento et al., 2021). Future studies using clinical examination are needed to assess the prevalence of some of the oral manifestations of COVID-19 such as hairy tongue and dental caries although this examination is best conducted in a clinic with precautions to prevention cross-infection. Third, reporting smoking may be underestimated, especially by females (Hwang et al., 2018). However, the under reporting is expected to be present in both COVID-infected and non-infected AYAs which would reduce the impact of this under-reporting on the observed association. Fourth, the snowball sampling strategy used with the electronic survey at the time of COVID-19 cannot guarantee statistical representativeness although all efforts were made to ensure maximum geographic coverage to increase generalizability. This method was also used previously in previous surveys conducted during the pandemic (Leighton et al., 2021).

## Conclusions

Smoking may confound the association between COVID-19 infection and the presence of some oral manifestations and modify the association with others. Policy makers, clinicians and health educators may need to take these associations into consideration when designing interventions.

## Supplemental Information

10.7717/peerj.13555/supp-1 Supplemental Information 1 The 43 countries where data were collected.

10.7717/peerj.13555/supp-2 Supplemental Information 2 Raw data.

10.7717/peerj.13555/supp-3 Supplemental Information 3 Questionnaire for the multi-country study.

## Additional Information and Declarations

### Data Availability

The following information was supplied regarding data availability: The raw data with codes and labels are available in the Supplemental File.
