# Supplementary material for: Oral manifestations in young adults infected with COVID-19 and impact of smoking: a multi-country cross-sectional study
Source: PeerJ. 2022 Jul 15;10:e13555. doi: 10.7717/peerj.13555 (PMC9291069; doi:10.7717/peerj.13555)
Supplement: Supplemental Information 1 [file peerj-10-13555-s001.docx]

**Appendix**

| **Country** | **N** | **%** |
| --- | --- | --- |
| 1. Algeria | 2 | <0.1 |
| 1. Argentina | 3 | 0.1 |
| 1. Australia | 3 | 0.1 |
| 1. Bahrain | 14 | 0.3 |
| 1. Belgium | 5 | 0.1 |
| 1. Canada | 37 | 0.7 |
| 1. Chad | 12 | 0.2 |
| 1. China | 4 | 0.1 |
| 1. Cyprus | 11 | 0.2 |
| 1. Egypt | 334 | 6.3 |
| 1. France | 8 | 0.1 |
| 1. Palestine | 56 | 1.0 |
| 1. Germany | 4 | 0.1 |
| 1. India | 454 | 8.5 |
| 1. Indonesia | 43 | 0.8 |
| 1. Iran | 5 | 0.1 |
| 1. Iraq | 88 | 1.6 |
| 1. Ireland | 3 | 0.1 |
| 1. Jordan | 52 | 1.0 |
| 1. Kuwait | 484 | 9.1 |
| 1. Lebanon | 31 | 0.6 |
| 1. Libya | 93 | 1.7 |
| 1. Malaysia | 126 | 2.4 |
| 1. Mauritania | 14 | 0.3 |
| 1. Morocco | 80 | 1.5 |
| 1. Holland | 3 | 0.1 |
| 1. Nigeria | 265 | 5.0 |
| 1. Oman | 174 | 3.3 |
| 1. Pakistan | 28 | 0.5 |
| 1. Philippines | 135 | 2.5 |
| 1. Qatar | 5 | 0.1 |
| 1. Saudi Arabia | 681 | 12.7 |
| 1. South Africa | 54 | 1.0 |
| 1. Sudan | 451 | 8.4 |
| 1. Sweden | 12 | 0.2 |
| 1. Syria | 326 | 6.1 |
| 1. Turkey | 596 | 11.2 |
| 1. Ukraine | 3 | 0.1 |
| 1. United Arab Emirates | 404 | 7.6 |
| 1. United Kingdom | 110 | 2.1 |
| 1. United States of America | 32 | 0.6 |
| 1. Yemen | 97 | 1.8 |
| Total | 53342 | 100.0 |
