# Supplementary material for: Oral manifestations in young adults infected with COVID-19 and impact of smoking: a multi-country cross-sectional study
Source: PeerJ. 2022 Jul 15;10:e13555. doi: 10.7717/peerj.13555 (PMC9291069; doi:10.7717/peerj.13555)
Supplement: Supplemental Information 3 [file peerj-10-13555-s003.docx]

**Oral lesions in young adults infected with COVID-19 and impact of smoking. A multi-country study**

You are invited to answer this questionnaire, which aims to identify the use of electronic cigarettes and cigarettes before and during the emerging coronavirus. Participation in the survey is not mandatory, and if you want to participate, answer the questions in the approval section. You can stop at any time by leaving the survey or closing the browser. The survey takes about 8-10 minutes. The data will be kept confidential and secure and can only be accessed by the research team.

For children under 18 years old, guardian consent is required, after which, the questionnaire can be given to the son / daughter to answer the questions themselves.

| **Age: □**11-14 **□**15-17 **□**18-23 | | | | | |
| --- | --- | --- | --- | --- | --- |
| I confirm that I have read and understood the information section of the participants in the current study. And I had the opportunity to absorb Information, asking questions and answering them satisfactorily.  I know that my participation is voluntary and that I am free to end my participation at any time by closing the survey or web browser without Showing any reason.  I agree to participate in this survey □Yes □No | | | | | |
| I am the parent of the son / daughter: I confirm that I have read and understood the information for the participants in the current study.  I know that my son / daughter's participation is voluntary and that he is free to end his participation at any time by closing the survey or the web browser without giving any reason.  Do you agree to participate in this questionnaire? □Agree □Disagree | | | | | |
| **Part I: Demographic and personal information:** | | | | | |
| **Gender:** □ Male □Female | | **Country of residence?**  **------------------------** | | **Do you have any medical condition?** □Yes □No | |
| **Your level of education**  **□** Illiterate  **□** Primary\Intermediate  **□** High school  **□** University degree and higher | | **Maternal education**  **□** Illiterate  **□** Primary\Intermediate  **□** High school  **□** University degree and higher | | **Paternal education**  **□** Illiterate  **□** Primary\Intermediate  **□** High school  **□** University degree and higher | |
| **Number of household members** | | **□**1 □2 □ 3 □ 4 □ 5 □ 6 □ 7 □ 8 □ 9 □ ≥10 | | | |
| **Number of bedrooms (except the kitchen and bathrooms)** | | **□**1 □2 □ 3 □ 4 □ 5 □ 6 □ 7 □ 8 □ 9 □ ≥10 | | | |
| **Part II: COVID-19 psychological effect and infection:** | | | | | |
| **Have you been infected with COVID-19** | | | | | □Yes □No |
| **Have you been suspected to be infected with COVID-19** | | | | | □Yes □No |
| **Have anyone of your family members or friend been infected with COVID-19** | | | | | □Yes □No |
| \| **12. During the last 2 weeks, how often you were anxious of the following** \| **Not at all** \| **Several days** \| **More than half the days** \| **Nearly Every day** \| \| --- \| --- \| --- \| --- \| --- \| \| \| Feeling nervous, anxious or on edge \| \| --- \| \|  \|  \|  \|  \| \| \| Not being able to stop or control worrying \| \| --- \| \|  \|  \|  \|  \| \| \| Worrying too much about different things \| \| --- \| \|  \|  \|  \|  \| \| \| Trouble relaxing \| \| --- \| \|  \|  \|  \|  \| \| \| Being so restless that it is hard to sit still \| \| --- \| \|  \|  \|  \|  \| \| Becoming easily annoyed or irritable \|  \|  \|  \|  \| \| Feeling afraid as if something awful might happen \|  \|  \|  \|  \| | | | | | |
| **Part III: smoking/ Vaping** | | | | | |
| 1. **Environmental tobacco and vaping distribution** | | | | | |
| **Are the following regular cigarette smokers, please select?** | | | □Mother □Father □Sibling □Close friends □None | | |
| **What is the percentage of your friends who are regular cigarette smokers?** | | | □ Non □ <25% □25%-50% □>50% | | |
| **How often does anyone smoke inside your home?** | | | □Daily □Weekly □Monthly □ Less Than Monthly □Never | | |
| **Are the following electronic cigarette user, please select?** | | | □Mother □Father □Sibling □Close friends □None | | |
| **What is the percentage of your friends who are electronic cigarette users?** | | | □ None □ <25% □ 25-50% □>50% | | |
| **How often does anyone use electronic cigarette inside your home?** | | | □ Daily □Weekly □Monthly □ Less Than Monthly □Never | | |
| 1. **Smoking\electronic cigarettes characteristics During COVID-19** | | | | | |
| **Do you smoke cigarettes?**  □Current smoker □ Former smoker □Never | | | **Who introduced you to regular cigarette?** □Parents  □friends □media/social media □advertisements □other □None | | |
| **number of cigarette/ day:**  □<5 □5-9 □10-14 □ 15-24 □>25 | | | **What is the frequency of using cigarettes?**  **□**Daily □Weekly □Monthly □less than monthly | | |
| **How did the smoking habits change during COVID-19?**  □ Less than before COVID-19  □Same as before COVID-19  □ More than before COVID-19 | | | **Did your frequency of night smoking increase after COVID-19?**  □Yes  □ No | | |
| **Do you use electronic cigarettes?**  □Yes □ No | | | **Who introduced you to electronic cigarette?** □Parents □friends □media/social media □ advertisements □ other □None | | |
| **number of e electronic cigarettes per day**  □0 □1 □2 □3 □4 □5 | | | **number of vaping puffs per use**  □1-2 □3-5 □4-6 □7-9 □$\geq$10 | | |
| **What is the frequency of using electronic cigarettes?**  **□**Daily □Weekly □Monthly □less than monthly | | |  | | |
| **How did your use of electronic cigarettes change during COVID-19?**  □More than before COVID-19  □Same as before COVID-19  □Less than before COVID-19 | | | **Did your use of night-time electronic cigarettes increase?**  □Yes  □ No | | |
| 1. **Attitude toward electronic cigarette and knowledge to its harmful effect** | | | | | |
| **Do you think electronic cigarette help one to quit cigarette smoking?** | | | □Agree □ Neutral □ Disagree | | |
| **Can electronic cigarettes cause addiction?** | | | □ Agree □ Neutral □ Disagree | | |
| **Should electronic cigarettes be prohibited for children below 18 years?** | | | □ Agree □ Neutral □ Disagree | | |
| **Are electronic cigarettes users more likely to be infected with COVID-19?** | | | □ Agree □ Neutral □ Disagree | | |
| **Are electronic cigarettes users more likely to have more severe symptoms of COVID-19 if infected?** | | | □ Agree □ Neutral □ Disagree | | |
| **Can electronic cigarettes be used in the treatment of COVID-19?** | | | □ Agree □ Neutral □ Disagree | | |
| **Do you think smoking\vaping can reduce anxiety caused by COVID-19?** | | | □ Agree □ Neutral □ Disagree | | |
| **Does electronic cigarettes have a harmful effect on the body?** | | | □ Agree □ Neutral □ Disagree | | |
| **Do you think you have the power to control your actions?** | | | □ Agree □ Neutral □ Disagree | | |
| **Do you have any of the following symptoms?** | | | | | |
| □None  □Teeth Discoloration  □ Dental Caries  □Gum inflammation  □White lesion | □Hairy tongue  □Taste alteration  □Vomiting /Nausea  □ Wound burns | | □ Mouth dryness  □ Mouth airway irritation  □ Cancer | | |
